# Synergistic Apoptotic Effects of Tocotrienol Isomers and *Acalypha wilkesiana* on A549 and U87MG Cancer Cells

**DOI:** 10.21315/tlsr2018.29.1.15

**Published:** 2018-03-02

**Authors:** Ibrahim Babangida Abubakar, Su-Wen Lim, Hwei-San Loh

**Affiliations:** 1 School of Biosciences, Faculty of Science, The University of Nottingham Malaysia Campus, 43500 Semenyih, Malaysia; 2 Biotechnology Research Centre, The University of Nottingham Malaysia Campus, 43500 Semenyih, Selangor, Malaysia

**Keywords:** Tocotrienol, Apoptosis, *Acalypha wilkesiana*, Synergism

## Abstract

Recent studies suggested that combined treatment approaches can be used to improve anticancer potency and circumvent the limitations of high-dose tocotrienols administration. *Acalypha wilkesiana* is a medicinal plant that has been used as an adjunct treatment for cancers in traditional medicine. Herein, the effects of single and combined treatments of β-, γ- and δ-tocotrienols and ethyl acetate extract (9EA) of *Acalypha wilkesiana* on lung (A549) and brain (U87MG) cancer cells were investigated. γ- and δ-tocotrienols exhibited higher potent antiproliferative effects against A549 (12.1 μg/ml and 13.6 μg/ml) and U87MG cells (3.3 μg/ml and 5.2 μg/ml) compared to β-tocotrienols (9.4 μg/ml and 92.4 μg/ml), respectively. Whereas, 9EA induced potent antiproliferative effects against U87MG cells only (2.0 μg/ml). Combined treatments of tocotrienols and 9EA induced a synergistic growth inhibition with up to 8.4-fold reduction in potent doses of β-, γ- and δ-tocotrienols on A549 cells. Apoptotic features were also evidenced on A549 cells receiving single and combined treatments. The synergism may greatly improve the therapeutic outcome for lung cancer.

## Abstract (ru)

Kajian terbaru mencadangkan bahawa pendekatan gabungan rawatan boleh digunakan untuk meningkatkan potensi antikanser dan menghindari batasan pemberian tocotrienol dos tinggi. *Acalypha wilkesiana* adalah tumbuhan ubatan yang telah digunakan sebagai rawatan tambahan untuk kanser dalam perubatan tradisional. Di sini, kesan rawatan tunggal dan gabungan β-, γ-dan δ-tocotrienols dan ekstrak etil asetat (9EA) daripada *Acalypha wilkesiana* pada paru-paru (A549) dan sel-sel kanser otak (U87MG) telah disiasat. γ-dan δ-tocotrienols menunjukkan kesan antiproliferatif yang lebih tinggi terhadap A549 (12.1 μg/ml dan 13.6 μg/ml) dan sel U87MG (3.3 μg/ml dan 5.2 μg/ml) berbanding β-tocotrienols (9.4 μg/ml), masing-masing. Sedangkan, 9EA merangsang kesan antiproliferatif yang kuat terhadap sel U87MG sahaja (2.0 μg/ml). Rawatan terapi tocotrienols dan 9EA mencetuskan perencatan pertumbuhan sinergis sehingga pengurangan 8.4 kali ganda dalam dos yang kuat dari β-, γ-dan δ-tocotrienols pada sel A549. Ciri-ciri apoptotik juga dibuktikan pada sel-sel A549 yang menerima rawatan tunggal dan gabungan. Sinergi ini boleh meningkatkan hasil terapeutik untuk kanser paru-paru.

## INTRODUCTION

Cancer is a group of disease caused by internal and external factors characterised by uncontrolled growth and spread of abnormal cells, resultant predominantly of dysregulation of cellular signalling. Cancer is the second major cause of death after cardiovascular diseases in the United States. In 2015, a total of 1,658,370 new cases and 589,430 deaths are expected (Siegel *et al.* 2015). Medicinal plants have continuously been explored as potential sources of new anticancer drugs. In fact, over 60% of approved chemotherapeutic agents are derived from natural sources. However, drug resistance and toxicity to non-cancerous cells have limited the potential application of chemotherapeutic agents. Tocotrienols are a group of vitamin E isomers isolated from non-medicinal oil palm plants with remarkable anticancer potency. However, the potency has been limited owing to high dosage which leads to metabolic degradation and subsequent reduction in available therapeutic doses (Shirode & Sylvester 2010). Synergistic drug combinations which are especially aimed at reducing dosage with improved potency could circumvent these limitations (Constantinou *et al.* 2008).

On the other hand, *Acalypha wilkesiana* is a medicinal plant used as a powdered mixture with other plants for treatment of breast cancer. Studies have reported that *A. wilkesiana* possesses several bioactivities including cytotoxicity against human cancers (Büssing *et al.* 1999; Lim *et al.* 2011). In fact, we recently showed that *A. wilkesiana* could be used as an adjunct treatment against brain and lung cancers (Lim *et al.* 2013). Hence, in this study, we investigated the synergistic potency of the combined treatments of tocotrienol isomers and the most potent ethyl acetate extract of *A. wilkesiana* (9EA) at lower dose, i.e. its minimum inhibitory concentration (MIC).

## MATERIALS AND METHODS

### Cell Culture Conditions and Plant Sample Preparation

Human lung adenocarcinoma (A549), glioblastoma (U87MG) and lung fibroblast (MRC5) cells were purchased from the American Type Culture Collection (ATCC, USA) and maintained in cell culture conditions as previously described (Abubakar *et al.* 2016). The collection and extraction details of the plant species, *A. wilkesiana* (voucher number: UNMC9) had been previously described (Lim *et al.* 2011). The most potent ethyl acetate extract of the *A. wilkesiana* whole plant (named as 9EA thereafter) tested by Lim and co-workers (Lim *et al.* 2011) was used in the study. Tocotrienol isomers (β, γ, δ) were supplied in kind by Davos Life Sciences Pte Ltd, Singapore. A stock solution of 100 mg/ml in dimethyl sulfoxide (DMSO) was prepared for 9EA*.* For tocotrienol isomers, a final stock concentration of 42.5 μg/ml was prepared as previously described (Lim *et al.* 2014b).

### Cell Viability Studies

A total of 5 × 10^3^ A549, U87MG and MRC5 cells were seeded in 96-well plates (SPL Life Sciences, Korea) and incubated overnight to facilitate attachment. Cells were treated with β-, γ- and δ-tocotrienols (0.4–42.5 μg/ml) and 9EA (0.1–1000 μg/ml) individually and incubated for 72h. For combined treatments, the minimum inhibitory concentration (MIC) of 9EA (0.1 μg/ml) was combined with these doses (0.4–42.5 μg/ml) of tocotrienols. On the other hand, cells receiving plain media with DMSO were served as untreated control. The cell viability was determined using the neutral red uptake assay according to a previously described protocol (Lim *et al.* 2014b). The IC_50_ values were determined using the nonlinear regression curve fit of the GraphPad Prism 5 software and results were presented as mean ± standard error of mean (SEM) of triplicates obtained from three independent experiments. Analysis of variance using completely randomised design was used to compare between treatment groups and the level of significance was set at p < 0.05.

### Determination of Synergism and Dose Reduction Index

The pharmacological interaction between potent tocotrienol isomers and 9EA was determined using the combinational index (CI) method as previously described (Wali & Sylvester 2007). The fold decrease in potent doses of β-, γ- and δ-tocotrienols and 9EA of the combined treatments was determined accordingly to the established dose reduction index (DRI) method (Wali & Sylvester 2007).

### Morphological Assessment of Apoptosis

Guided by the cell viability data, 5 × 10^3^ A549 cells were seeded in 2-well chamber slides (SPL Life Sciences, Korea) and treated with IC_50_ doses or combined lower doses of tocotrienols with MIC of 9EA for 72h. Cells treated with plain media containing DMSO served as untreated controls. Changes on the cellular morphology were observed by using fluorescence dyes, namely the acridine orange (Nacalai Tesque, Japan) and propidium iodide (Invitrogen, USA) which were prepared accordingly to the described protocol (Lim *et al.* 2013) under an epifluorescence microscope (Nikon, Japan).

## RESULTS

In the present study, the antiproliferative effects of three different tocotrienol isomers comprising β-, γ- and δ-tocotrienols were tested on human lung (A549) and brain (U87MG) cancer cells. Results from cell viability test showed that γ-, and δ-tocotrienol isomers exhibited greater potency especially towards U87MG cells with IC_50_ values of 3.3 μg/ml and 5.2 μg/ml, respectively (Table 1). Similar growth inhibitory effects were also evident on 9EA-treated U87MG cells with IC_50_ value below the 20 μg/ml potency level for plant extract as established by the national cancer institute (NCI) (Suffness & Pezzuto 1990). In contrast, less toxicity was evident on A549 cells (IC_50_ value of 90 μg/ml) treated with 9EA (Table 1). Meanwhile, both tocotrienols and 9EA were also tested on normal lung fibroblast (MRC5) cells for comparison purpose. As shown in Table 1, the IC_50_ values obtained were above 200 μg/ml indicating non-toxic effects towards the non-cancerous cells as opposed to cancer cells. Guided by CI values of less than 1 (Table 2), combined treatments of 0.1 μg/ml (MIC) of 9EA with β-, γ-, and δ-tocotrienols synergistically inhibited the proliferation of A549 cells with considerable fold reduction in the required potent dose for tocotrienol isomers. The greatest synergism was evident on A549 cells receiving the combined low-dose treatment of δ-tocotrienol with MIC dose of 9EA causing up to 8.4-fold reduction of potent δ-tocotrienol dose. In contrast, although single treatments of β-, γ-, and δ-tocotrienols and 9EA were evident possessing more potent effects on U87MG than A549 cells, synergism was only found on such U87MG cancer cells receiving the combined treatment of δ-tocotrienol and 9EA (CI = 0.20) as shown in Table 2. Pleasingly, the combined treatments of tocotrienols with MIC of 9EA did not induce toxic effects on non-cancerous MRC5 cells with IC_50_ values above 200 μg/ml for tocotrienols (Table 3) in contrast to toxicity demonstrated by previously tested control drug, vinblastine (Lim *et al.* 2011).

**Figure 1:**
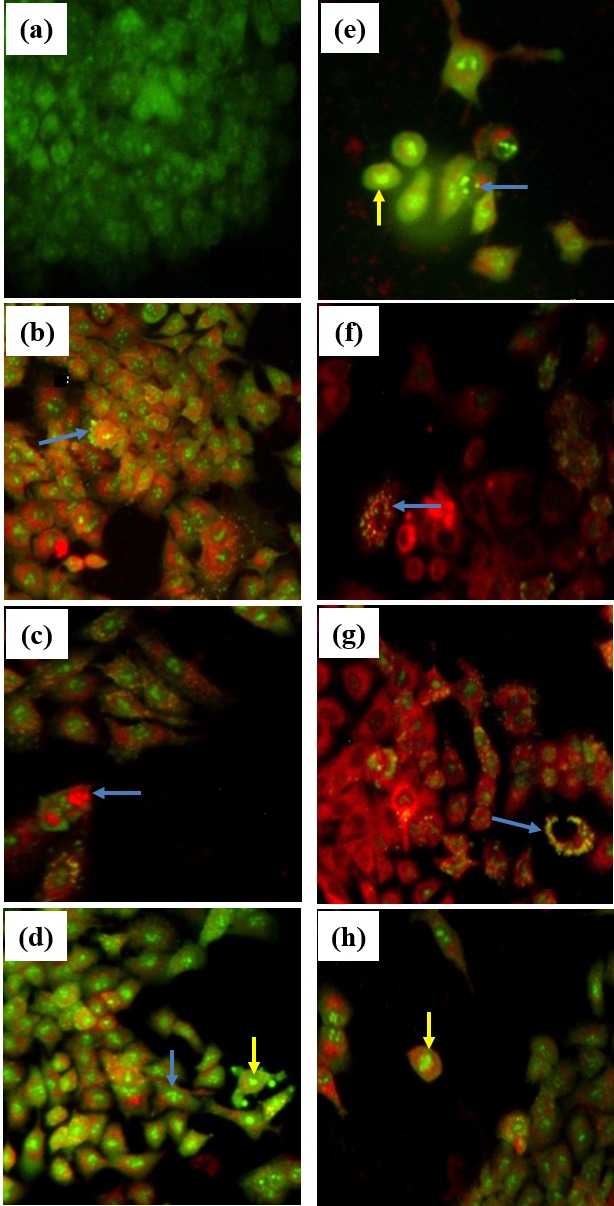
Representative merged AO/PI images of A549 cells treated with individual IC_50_ doses of tocotrienols and *A. wilkesiana* 9EA as well as combined low-dose treatments of tocotrienols with MIC of 9EA (0.1 μg/ml). Morphologies of (a) untreated cells, cells receiving treatments of (b) δ-tocotrienol (13.6 μg/ml), (c) γ-tocotrienol, (12.1 μg/ml), (d) β-tocotrienol (92.4 μg/ml), (e) 9EA (90.3 μg/ml), (f) δ-tocotrienol + 9EA MIC (1.6 + 0.1 μg/ml), (g) γ-tocotrienol + 9EA MIC (5.0 + 0.1 μg/ml), (h) β-tocotrienol + 9EA MIC (12.0 + 0.1 μg/ml) are shown. Morphological features of apoptosis including nuclear chromatin condensation (yellow arrows) and chromatin fragmentation (blue arrows) are evident with majority cells stained either red or orange indicating a later stage of apoptosis. Images were captured at 40× magnification under the epifluorescence microscope (Nikon, Japan).

The effect of single and combined treatments on the morphology of A549 cells was also evaluated. As shown in Fig. 1, both individual IC_50_ doses and combined low-dose treatment of tocotrienols with 9EA MIC caused changes in cellular morphology with apoptotic features evidenced including the chromatin condensation and nuclear chromatin fragmentation. The merged AO/PI images for single and combined treatments stained either in red or orange indicating a later stage of apoptosis. These are consistent with the previously described morphological features of apoptosis (Saraste & Pulkki 2000).

## DISCUSSION

Natural products from plants are continuously developed as potential anticancer agents due to the history of medicinal uses of plants as well as lower side effects as compared to synthetic drugs. However, drug resistance and low availability of therapeutic dose have limited the potency of some anticancer candidates such as tocotrienols. Combination treatment approach has persistently been explored as an alternative for improving potency of tocotrienols (Akl *et al.* 2012; Sylvester *et al.* 2010; Abubakar *et al.* 2014). Herein, the antiproliferative potency can be ranked in a descending order as δ->γ->β-tocotrienols, conforming to the findings of previous studies (Constantinou *et al.* 2008; Lim *et al.* 2014b). On the other hand, the selection of *A. wilkesiana* in the current study was rationalised based on a previous suggestion which had stated its feasibility in the combined treatment (Lim *et al.* 2013). Furthermore, evidence from traditional medicine showed that the powdered mixture of *A. wilkesiana* combined with four other plants has been used in southwest Nigeria for treating breast cancer (Büssing *et al.* 1999). In the present study, 9EA exhibited potent antiproliferative effects against U87MG only but not A549 cells. This is in line with the previous findings on *A. wilkesiana* 9EA (Lim *et al.* 2011).

Drug resistance and low bioavailability of therapeutic doses limit the potency of chemotherapeutic drugs *in vivo* and in clinical trials. For instance, orally delivered high-dose tocotrienol is associated with metabolic degradation and low therapeutic dose that jeopardise its potential in cancer treatment (Shirode & Sylvester 2010). As such, following the antiproliferative screening of individual doses of tocotrienols and 9EA, the lower dose at MIC of 9EA was combined with tocotrienols in hope to minimise the required potent doses and improve the potency of tocotrienols. Indeed as shown in Table 2, combined low-dose treatments induced synergistic growth inhibition against A549 cells with considerable reduction of up to 8.4 and 900 folds for potent tocotrienol isomers (β, γ, δ) and 9EA doses, respectively. In contrast, only combined low-dose treatment of δ-tocotrienol with 9EA (MIC) induced synergistic antiproliferative affects against U87MG cells. This is surprising as single treatment of 9EA induced higher potent effects against U87MG (IC_50_ = 2.0 μg/ml) than A549 cells (IC_50_ = 90.3 μg/ml). The potency of 9EA as single treatment and in combination with tocotrienols might be affected differently by the types of cancer cells tested. For instance, γ-tocotrienol triggered caspase dependent and independent apoptosis in prostate cancer cells (Constantinou *et al*. 2012, 2009) in contrast to activation of caspase dependent pathway which was only occurred in murine mammary cancer cells (Shah *et al.* 2003). Furthermore, considering that 9EA contains numerous bioactive compounds in the extract which could also account for the observed difference in potency for single doses of 9EA. Previous studies have demonstrated that the potencies and mechanisms of action of chemotherapeutic agents are dependent on a cellular microenvironment. Hence, the type of pharmacological interaction for combined low-dose treatment with tocotrienols is largely dependent on the microenvironments of A549 and U87MG cells. The present results suggest that whilst single treatment of 9EA possesses a higher potency against U87MG brain cancer, the combined low-dose treatment with tocotrienols is however most suitable for treatment against A549 lung cancer.

The present study has corroborated previous findings that demonstrated the synergistic cytotoxic potency of tocotrienols. For instance, the combined treatment of δ-tocotrienol with jerantinine B caused up to 2-fold reduction of potent δ-tocotrienol doses and improved potency of tocotrienol via disruption of microtubules (Abubakar *et al*. 2016). Similarly, combined treatments of tocotrienols at lower dosages with alkaloid crude extract of *Ficus fistulosa*, sesamin and statins have effectively caused dose reduction and improved potency (Abubakar *et al.* 2014; Akl *et al*. 2012; Wali & Sylvester 2007). Unlike tocotrienols, very scarce studies have demonstrated the synergistic potency of *A. wilkesiana.* For instance, a previous study had stated that *A. wilkesiana* extract enhanced the potency of α-tocopherol (Lim *et al*. 2013) but without investigating the dose reduction index and type of pharmacological interaction between *A. wilkesiana and* α-tocopherol. Nonetheless, the present study has supported this previous finding on the synergistic potency of *A. wilkesiana.*

Considering more interesting CI values (Table 2) were obtained from A549 cells, further cellular morphological assessment on all combined low-dose treatments of tocotrienols with MIC of 9EA that induced synergistic antiproliferative effects in such cells was conducted. Apoptosis is characterized by morphological features that include cell shrinkage, nuclear chromatin condensation and fragmentation, membrane blebbing and formation of apoptotic bodies (Elmore 2007; Saraste & Pulkki, 2000). In this study, the combined low-dose treatments of tocotrienols with 9EA (MIC) induced chromatin condensation and fragmentation suggesting a possible induction of apoptosis in A549 cells. In fact, the confirmation of apoptosis induction by tocotrienol isomers (β, γ, δ) and 9EA had been separately reported (Lim *et al.* 2011, 2014a, 2014b).

## CONCLUSION

This study exhibited the antiproliferative potency of *A. wilkesiana* demonstrating its suitability as an adjunct treatment, thus supporting the common practice of combining *A. wilkesiana* with other plants for cancer treatment by traditional healers. Furthermore, the combined treatments with tocotrienols caused a reduction in required potent doses which would minimise toxicity to non-cancerous cells, thus reducing side effects; improve anticancer potency and potentially circumvent limitations associated with high-dose tocotrienols. This preliminary finding of bioactive synergism also provides an insight on the development of value-added chemotherapeutic regimen against lung cancer in future.

## Figures and Tables

**Table 1:** Antiproliferative effects of tocotrienol isomers and ethyl acetate extract of *A. wilkesiana* (9EA) on A549, U87MG and MRC5 cells.

No.	Treatment	A549	U87MG	MRC5

		Mean ± SEM, IC_50_ Values (μg/ml)		
1	β-Tocotrienol	92.4 ± 0.66	9.4 ± 0.05	>200.0#
2	γ-Tocotrienol	12.1 ± 0.11	3.3 ± 0.08	>200.0#
3	δ-Tocotrienol	13.6 ± 0.50	5.2 ± 0.07	>200.0#
4	9EA	90.3 ± 0.25	2.0 ± 0.16	>200.0#

The doses required to induce 50% cell growth inhibition (IC_50_) were determined after 72h using the neutral red uptake assay. The concentration range used was 0.4–42.5 μg/ml for tocotrienol isomers.

# IC_50_ value obtained was greater than the maximum dose.

**Table 2:** Combinational index (CI) and dose reduction index (DRI) of combined treatments of low-dose β-, γ- and δ-tocotrienols with MIC of 9EA on A549 and U87MG cells.

No.	Treatment	Tocotrienol (IC_50_, μg/ml)	9EA (MIC, μg/ml)	Combination index (CI)	Dose reduction index (DRI)	

					Tocotrienol	9EA
A549 cells						

1	β-Tocotrienol	12.0 ± 0.10	0.1	**0.13**	7.7 ± 1.26	900
2	γ-Tocotrienol	5.0 ± 0.21	0.1	**0.42**	2.4 ± 1.60	900
3	δ-Tocotrienol	1.6 ± 0.23	0.1	**0.12**	8.4 ± 1.69	900

U87MG cells						

4	β-Tocotrienol	13.2 ± 0.04	0.1	1.45	-	-
5	γ-Tocotrienol	9.1 ± 0.05	0.1	2.80	-	
6	δ-Tocotrienol	0.8 ± 0.09	0.1	**0.20**	6.6 ± 1.38	10

MIC of 9EA was combined with β-, γ- and δ-tocotrienols. The IC_50_ doses of tocotrienols following the combined treatments were determined after 72h using the neutral red uptake assay. CI values indicate synergism (< 1, **bolded**), antagonism (> 1) and additive (= 1) pharmacological interaction between tocotrienol isomers and *A. wilkesiana* 9EA.

**Table 3:** Antiproliferative effects of combined treatments of tocotrienols with MIC of 9EA on MRC5 cells.

No.	Treatment	Tocotrienol (IC_50_, μg/ml)	9EA (MIC, μg/ml)
1	β-Tocotrienol	200#	0.1
2	γ-Tocotrienol	200#	0.1
3	δ-Tocotrienol	200#	0.1

The IC_50_ values were determined after 72h using the neutral red uptake assay.

# IC_50_ value obtained was greater than the maximum dose.
